# From discordance to clinical decision support: integrating longitudinal imaging and ctDNA through multimodal artificial intelligence in precision oncology

**DOI:** 10.3389/fonc.2026.1963645

**Published:** 2026-10-27

**Authors:** Jian Zheng, Luyang Chi, Yong Guo

**Affiliations:** Department of Oncology, Zhejiang Provincial Hospital of Chinese Medicine, The First Affiliated Hospital of Zhejiang Chinese Medical University, Hangzhou, Zhejiang, China

**Keywords:** artificial intelligence, circulating tumor DNA, clinical decision support, discordance, liquid biopsy, multimodal integration, precision oncology

## Abstract

Accurate treatment-response assessment increasingly requires information that is spatial, molecular, and longitudinal. Imaging depicts lesion burden, anatomical distribution, and morphological evolution, whereas circulating tumor DNA (ctDNA) provides a systemic molecular signal shaped by tumor shedding, clonal composition, assay characteristics, and treatment pressure. Because these modalities measure non-identical dimensions, disagreement between them is clinically plausible and can reflect lead-lag kinetics, site-specific progression, clonal evolution, or technical uncertainty. In this focused narrative review, we synthesize evidence for imaging-ctDNA integration and propose a multidimensional, multilabel taxonomy of discordance spanning directional, temporal, spatial, molecular, and technical dimensions. We distinguish isolated from persistent discordance and emphasize exact temporal alignment, analytical confidence, and orthogonal confirmation. Current evidence supports prognostic complementarity and demonstrates measurable discordance across tumor types, but clinical actionability is context dependent. Randomized studies such as PADA-1 show that molecular-first treatment adaptation can be beneficial when a defined biomarker is linked to a validated intervention, whereas other trials illustrate that molecular detectability alone does not guarantee benefit from early treatment. We therefore frame multimodal artificial intelligence (AI) as an uncertainty-aware decision-support approach rather than an automated treatment selector. Future systems should preserve modality-specific information, handle irregular longitudinal sampling, quantify uncertainty, identify information gaps, and recommend evidence-matched reassessment. Prospective validation should prioritize clinical utility, harms, transportability, governance, and net benefit rather than discrimination alone.

## 1 Introduction

Accurate and timely assessment of treatment response is fundamental to precision oncology. Conventional imaging remains indispensable because it localizes disease, quantifies lesion burden, and reveals organ-specific patterns such as oligoprogression, mixed response, and sanctuary-site progression. Yet anatomical change is an indirect and sometimes delayed consequence of tumor biology, and treatment-related inflammation, necrosis, fibrosis, or cavitation can complicate interpretation.

ctDNA provides a complementary view. Serial plasma measurements can capture changes in tumor-derived molecular burden, molecular residual disease, and emerging resistance-associated variants. However, ctDNA is not a direct substitute for imaging. Detectability depends on disease burden, metastatic site, vascularity, biological phenotype, treatment phase, assay design, preanalytical handling, and the analytical limit of detection. A negative result therefore does not establish absence of active disease.

These differences make imaging-ctDNA discordance an expected feature of longitudinal monitoring rather than a single error state. The clinically important question is not which modality is universally correct, but what type of disagreement is present, how confident the observation is, what alternative technical explanations remain, and whether additional information can reduce uncertainty.

Prior reviews have examined the integration of imaging and ctDNA for outcome prediction and the broader use of artificial intelligence for multimodal data integration in oncology (1, 2). The present review focuses more narrowly on discordance itself. We synthesize quantitative and interventional evidence, propose an operational multidimensional taxonomy, separate molecular detectability from prognostic validity and treatment actionability, and define requirements for future discordance-aware multimodal AI.

A central premise of this review is that AI should not be treated as a mechanism for converting heterogeneous inputs into a single response label. Its more defensible near-term role is to preserve modality-specific signals, recognize discordant trajectories, quantify uncertainty, identify missing information, and support reassessment. Treatment modification should remain linked to disease-specific evidence and clinician judgment.

## 2 Literature search and evidence selection

This focused narrative review was informed by a structured literature search. PubMed/MEDLINE was searched from database inception through 17 September 2026. Search concepts combined terms for circulating tumor DNA and liquid biopsy (for example, “circulating tumor DNA,” “ctDNA,” “cell-free DNA,” “liquid biopsy,” and “molecular residual disease”) with imaging terms (“imaging,” “radiology,” “radiomics,” “CT,” “MRI,” “PET,” and “RECIST”), longitudinal monitoring terms (“treatment response,” “monitoring,” “longitudinal,” “progression,” “discordance,” and “concordance”), and multimodal AI terms (“multimodal,” “artificial intelligence,” “machine learning,” “deep learning,” “data integration,” and “fusion”). Additional targeted searches of publisher and regulatory sources addressed biomarker-guided treatment adaptation, molecular progression, assay standardization, AI reporting, and clinical deployment.

Priority was given to original clinical studies in which imaging and circulating molecular biomarkers were evaluated within the same population; longitudinal studies with serial imaging or ctDNA; studies reporting concordance or discordance; multimodal prediction studies; prospective biomarker-guided studies; randomized trials relevant to molecular-first actionability; and authoritative methodological or regulatory guidance. Reference lists of eligible primary studies and relevant reviews were screened for additional sources. This was not designed as a systematic review or meta-analysis; therefore, no pooled effect estimate was calculated. Negative statements regarding the maturity or absence of discordance-aware systems are restricted to the literature identified within this search framework.

The search was updated on 17 September 2026. Because this article is a focused narrative review rather than a systematic review, the search was used to structure evidence identification and verification rather than to generate a PRISMA-style exhaustive study set.

## 3 Imaging-ctDNA discordance: from observation to an operational taxonomy

### 3.1 Complementary biological dimensions

Imaging and ctDNA measure related but non-identical aspects of cancer. Imaging is spatially resolved and lesion centered; ctDNA is a systemic molecular signal weighted toward lesions that shed DNA into the circulation. Imaging can identify where progression occurs but cannot directly define the molecular clone responsible. ctDNA can reveal molecular evolution but usually cannot localize the anatomical source of that signal. This non-equivalence is the biological basis for both concordance and discordance. The overall conceptual relationship between complementary imaging and ctDNA measurements, discordance classification, and uncertainty-aware clinical decision support is illustrated in **Figure 1**.

**Figure 1:**
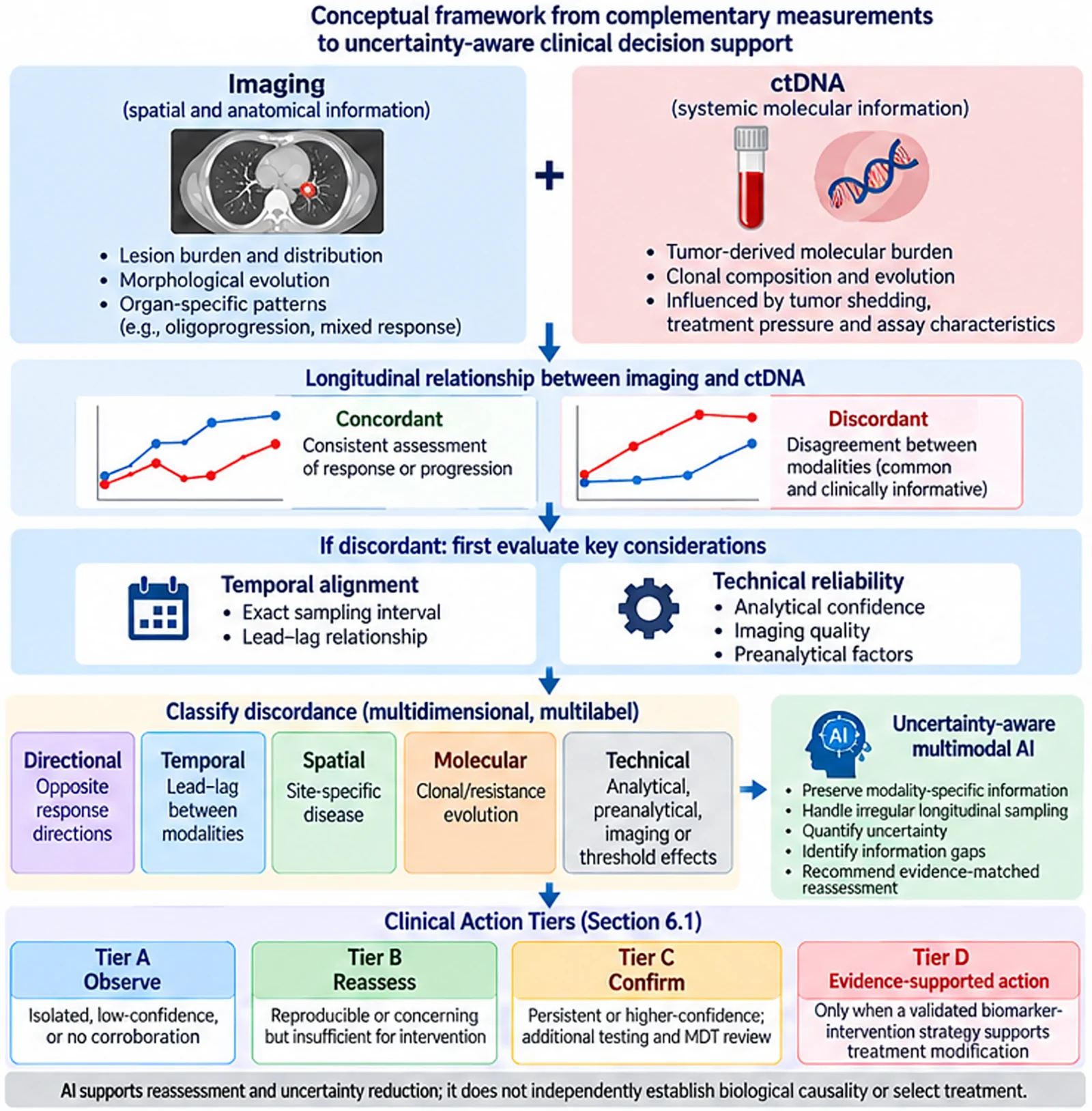
Conceptual framework from complementary measurements to uncertainty-aware clinical decision support. Imaging provides spatial and anatomical information, whereas ctDNA provides systemic molecular information influenced by tumor shedding and clonal evolution. Their longitudinal relationship may be concordant or discordant. Discordant observations should first be evaluated for temporal alignment and technical reliability, then classified across directional, temporal, spatial, molecular, and technical dimensions. Multimodal AI is positioned as a decision-support layer that quantifies uncertainty and identifies the next assessment most likely to reduce uncertainty; it does not independently establish biological causality or select treatment. AI, artificial intelligence; ctDNA, circulating tumor DNA. Initial schematic design was assisted by OpenAI ChatGPT and was reviewed and edited by the authors.

### 3.2 Technical and biological competing explanations

A single discrepant observation should initially be treated as an unresolved cross-modal signal rather than proof of occult progression or resistance. For ctDNA, relevant sources of uncertainty include plasma input, preanalytical handling, sequencing depth, background-error suppression, assay limit of detection or quantification, tumor-informed versus tumor-naive design, clonal hematopoiesis, low-shedding disease, transient treatment-related DNA release, and the distinction among variant allele frequency, absolute ctDNA concentration, tumor fraction, and binary detectability (3, 4). For imaging, uncertainty arises from acquisition and reconstruction protocols, contrast timing, segmentation, lesion selection, response criteria, treatment-related change, and inter-reader variability (5). Differences in assay design, analytical sensitivity, tumor fraction estimation, and biological shedding mean that an undetectable or low-level ctDNA result during treatment should be interpreted within the specific assay and clinical context rather than as a universal biological state (6, 7).

### 3.3 Proposed multidimensional, multilabel taxonomy

We propose five dimensions: directional, temporal, spatial, molecular, and technical discordance. These dimensions are not mutually exclusive. A patient can simultaneously exhibit directional discordance (opposite response directions), temporal discordance (lead-lag between modalities), molecular discordance (emergence of a resistance-associated clone), and spatial discordance (a lesion whose behavior is incompletely represented in plasma). The framework should therefore be applied as a multilabel description rather than as a single subtype. The proposed operational dimensions of discordance are summarized in **Table 1**, while their multilabel relationships, cross-cutting modifiers, and corresponding Clinical Action Tiers are illustrated in **Figure 2**.

**Table 1:** Proposed operational framework for multidimensional imaging-ctDNA discordance.

Dimension	Operational definition	Representative pattern	Required information	Major alternative explanation	Proposed interpretation
Directional	Imaging and ctDNA change in different directions within a prespecified assessment window.	RECIST SD/PR with reproducible ctDNA rise; or radiographic PD with falling/undetectable ctDNA.	Exact dates; imaging category/quantitative change; serial ctDNA metric and assay.	Measurement variability; transient ctDNA release; pseudo progression; asynchronous sampling.	Reproduce the signal and assess technical confidence before biological attribution.
Temporal	Modalities ultimately change in the same direction but at different times.	ctDNA rise precedes radiographic progression; ctDNA clearance precedes shrinkage.	At least two longitudinal assessments where available; exact lead/lag interval.	Different sampling cadence or delayed anatomical remodeling.	Report lead/lag explicitly; do not label asynchronous observations as strictly contemporaneous.
Spatial	Lesion- or organ-specific progression is incompletely represented by systemic ctDNA.	CNS, local, pulmonary, or low-volume progression with negative/declining ctDNA.	Lesion location, burden, organ distribution, imaging field, assay sensitivity.	Low total burden or low-shedding disease.	Treat modalities as spatially non-equivalent; consider site-specific assessment.
Molecular	ctDNA reveals clonal or resistance evolution not visible on conventional imaging.	Emerging ESR1 or other resistance-associated alteration during radiographic stability.	Variant identity, serial VAF/tumor fraction, assay design, biological relevance.	Clonal hematopoiesis, sequencing artifact, non-actionable subclone.	May justify confirmation or disease-specific action only when biomarker-intervention evidence exists.
Technical	Mismatch is plausibly explained by analytical, preanalytical, imaging, or threshold effects.	Low-level ctDNA oscillation near LOD; borderline lesion change near RECIST threshold.	LOD/LOQ, plasma volume, CHIP filtering, scanner/protocol, segmentation/reader information.	True early biological change remains possible.	Technical explanation is an explicit competing hypothesis, especially for isolated discordance.

**Figure 2:**
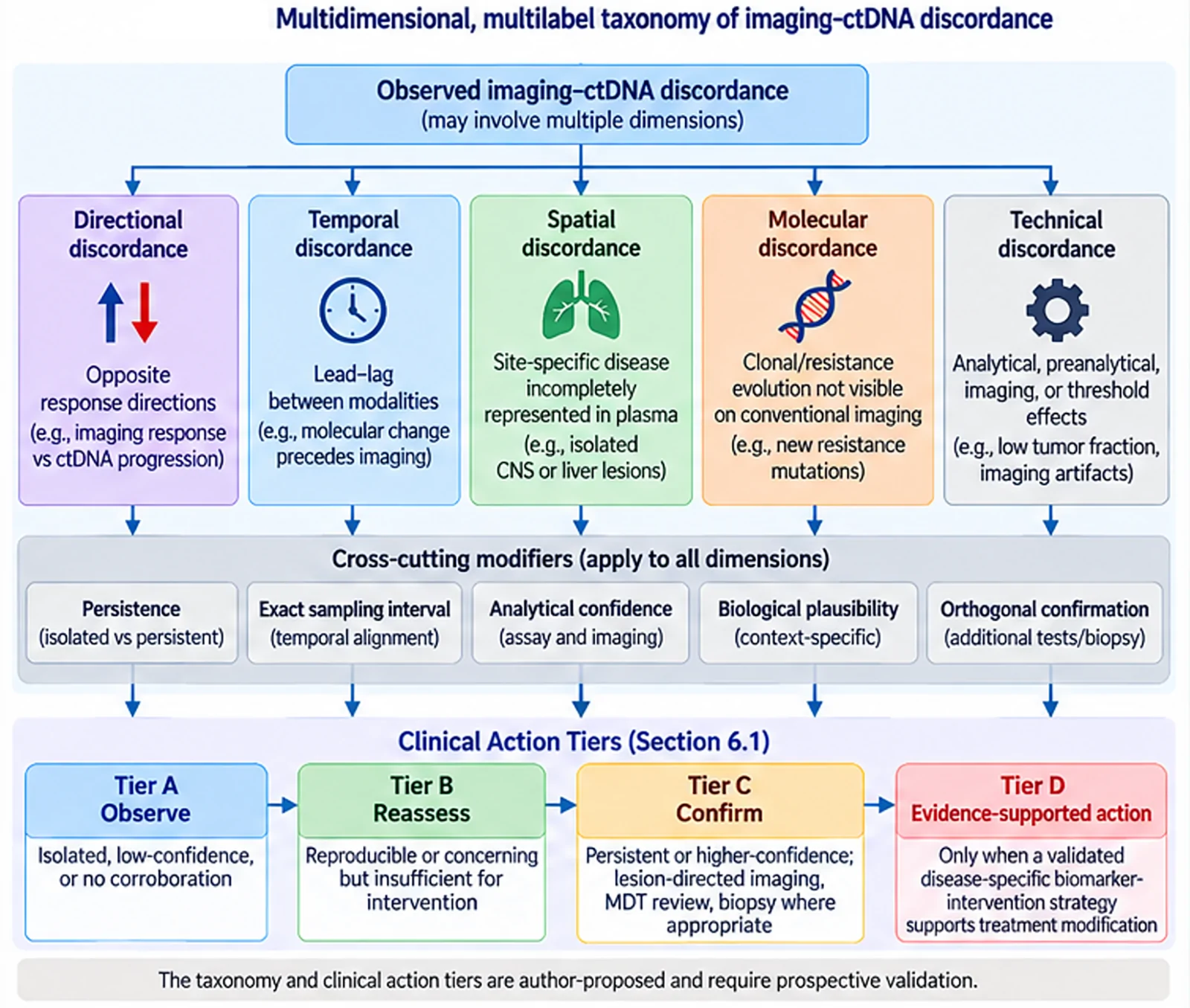
Multidimensional, multilabel taxonomy of imaging-ctDNA discordance. An observed imaging-ctDNA mismatch can receive one or more labels across five dimensions: directional, temporal, spatial, molecular, and technical. Cross-cutting modifiers include persistence, exact sampling interval, analytical confidence, biological plausibility, and orthogonal confirmation. Clinical responses are separated from taxonomy labels and summarized as Tier A (Observe), Tier B (Reassess), Tier C (Confirm), and Tier D (Evidence-supported action). The taxonomy and tiers are author-proposed and require prospective validation. Initial schematic design was assisted by OpenAI ChatGPT and was reviewed and edited by the authors.

### 3.4 Cross-cutting modifiers and operational definitions

For research standardization, we propose that isolated discordance denote a single discrepant observation not yet reproduced and persistent discordance denote a discrepant pattern confirmed on at least two sequential assessments using comparable methods. These are proposed operational definitions, not validated clinical thresholds. The exact interval between imaging and blood collection should always be reported. No universal acceptable interval can currently be justified across tumor types, treatment mechanisms, and assay platforms. Prospective studies should prespecify disease- and treatment-specific temporal windows; observations outside that window should be interpreted as asynchronous, with reduced confidence in strict contemporaneous discordance and explicit consideration of lead-lag effects.

Similarly, no universal percentage change in ctDNA should define molecular response or progression. Interpretation should be assay specific and should consider analytical variation, baseline abundance, reproducibility, tumor fraction, and the biological meaning of the tracked alteration.

### 3.5 Quantitative evidence that discordance occurs

Discordance has been observed empirically, although reported frequencies are not directly poolable. In a cohort of 821 patients with nasopharyngeal carcinoma, MRI/RECIST-based response and post-neoadjuvant cfEBV-DNA response were discordant in 25.3% of patients (8). In a pilot cohort of advanced genitourinary malignancies treated with immune checkpoint inhibitors, ctDNA dynamics and imaging were concordant in 15 of 18 evaluable patients (83%); among three discordant cases, two later developed central nervous system metastases and one developed extracranial progression despite persistently undetectable ctDNA (9). In postoperative pancreatic ductal adenocarcinoma surveillance, imaging and ctDNA were concordant in 39 of 50 patients (78%), whereas 11 were discordant; 10 of those 11 had radiologic recurrence with negative ctDNA (10).

These observations support the existence of clinically relevant cross-modal disagreement but also illustrate why no universal discordance rate should be quoted. Tumor type, disease burden, metastatic distribution, assay design, sampling interval, imaging schedule, and the operational definition of discordance differ substantially among studies.

Note: The taxonomy is author proposed and intended for prospective testing. Dimensions are multilabel rather than mutually exclusive. LOD, limit of detection; LOQ, limit of quantification; PD, progressive disease; PR, partial response; SD, stable disease; VAF, variant allele frequency.

## 4 Current clinical evidence for imaging-ctDNA integration and discordance

### 4.1 Prognostic complementarity does not equal clinical utility

Several studies support the premise that imaging and circulating molecular measurements contain nonredundant information. In metastatic non-small-cell lung cancer, a longitudinal ctDNA model derived from IMpower150 stratified overall survival even within RECIST-defined response categories: among patients with stable disease, median overall survival was approximately 7.1 versus 22.3 months (HR 3.2), and among those with partial response it was approximately 8.8 versus 28.6 months (HR 3.3) (11). This finding directly illustrates molecular heterogeneity within a common radiographic label.

In localized lung cancer treated with chemoradiotherapy, integration of radiomic information with mid-treatment ctDNA dynamics improved dynamic risk assessment (12). Such studies support cross-modal complementarity, but they should not be interpreted as evidence that AI-guided treatment changes improve outcomes.

A baseline multimodal study in resectable esophageal adenocarcinoma illustrates the same distinction. The best combined models achieved C-indices of approximately 0.55-0.59 compared with approximately 0.44-0.45 for the clinical comparator (13). These values demonstrate feasibility of joint modeling but modest discrimination; the study is therefore not presented as proof of clinical utility.

### 4.2 Quantitative discordance and site-specific limitations

The nasopharyngeal carcinoma, genitourinary immunotherapy, and pancreatic surveillance cohorts summarized above provide direct quantitative evidence that imaging and molecular response classifications can disagree. Importantly, the pancreatic and genitourinary examples also highlight spatial limitations of plasma ctDNA: clinically relevant progression may occur in sites or disease states that are poorly represented in blood.

### 4.3 Molecular detectability, prognostic validity, and treatment actionability

These concepts should be separated. PADA-1 provides randomized evidence that molecular-first treatment adaptation can be beneficial in a narrowly defined setting. Patients with advanced hormone-receptor-positive, HER2-negative breast cancer receiving an aromatase inhibitor plus palbociclib who developed a rising ESR1 mutation without synchronous progression were randomized to switch endocrine therapy or continue; the biomarker-guided switch improved progression-free survival (14). This establishes actionability for a specific biomarker-intervention pair, not for nonspecific ctDNA elevation or generic imaging-ctDNA discordance.

DYNAMIC similarly demonstrates that postoperative ctDNA can guide adjuvant treatment allocation in stage II colon cancer while reducing chemotherapy use without compromising recurrence-free survival (15). However, this is an MRD-guided adjuvant strategy rather than an imaging-ctDNA discordance intervention during systemic therapy. Large prospective observational data from the CIRCULATE-Japan GALAXY program further support the strong prognostic value of postoperative ctDNA positivity in resectable colorectal cancer (16).

Conversely, ALTAIR, embedded within CIRCULATE-Japan, randomized 243 patients with resected colorectal cancer who became ctDNA positive after standard therapy but had no radiological evidence of disease. Trifluridine/tipiracil did not significantly improve disease-free survival versus placebo (median 9.30 vs 5.55 months; HR 0.79, 95% CI 0.60-1.05; P = 0.107) (17). The result illustrates that molecular recurrence does not automatically make a particular early intervention beneficial.

Accordingly, molecular detectability, prognostic validity, and treatment actionability represent separate evidentiary steps. The most defensible general principle is not “ctDNA progression should trigger treatment,” but “a molecular signal can support action when the biomarker, disease context, and intervention have been prospectively validated.” Representative studies informing imaging-ctDNA integration, quantitative discordance, and biomarker-guided actionability are summarized in **Table 2**.

**Table 2:** Representative clinical evidence informing imaging-ctDNA integration, discordance, and biomarker-guided actionability.

Study/setting	Population	Imaging/clinical comparator	Molecular approach	Key finding	Relevance	Main limitation
IMpower150 longitudinal model	Metastatic NSCLC; n=466	RECIST response	Serial ctDNA at 5 time points	OS stratification within SD and PR groups (HR ~3.2-3.3)	Molecular trajectories add information within radiographic categories	Prognostic; not intervention guided
NPC cfEBV-DNA study	Nasopharyngeal carcinoma; n=821	MRI/RECIST after neoadjuvant therapy	Post-treatment cfEBV DNA	25.3% discordance between radiologic and biological response	Direct quantitative discordance evidence	EBV-specific biomarker and disease
Advanced GU ICI cohort	20 patients; 18 evaluable	Conventional imaging	Longitudinal ctDNA	83% concordance; 3 discordant, including 2 later CNS metastases	Illustrates spatial discordance/low plasma representation	Small pilot cohort
Localized NSCLC radiomics-ctDNA	Chemoradiotherapy; n=418	Serial imaging/radiomics	Mid-treatment ctDNA dynamics	Improved dynamic risk stratification	Supports longitudinal complementarity	No proof of model-guided clinical benefit
PDAC surveillance	Resected PDAC; n=50	CT or CT/MRI surveillance	Tumor-specific ctDNA	78% concordance; 22% discordance; 10 radiologic recurrences were ctDNA negative	Site-specific limits of plasma surveillance	Single-center retrospective
PADA-1	Advanced hormone receptor-positive/HER2-negative breast cancer	No synchronous progression at molecular trigger	Rising ESR1 mutation	Biomarker-guided endocrine switch improved PFS	Prospective actionability of a defined biomarker-intervention pair	Not generalizable to nonspecific ctDNA or AI discordance
DYNAMIC	Stage II colon cancer; randomized	Standard clinicopathologic management	Postoperative ctDNA	Reduced adjuvant chemotherapy use without compromising RFS	Prospective ctDNA-guided management	MRD/adjuvant setting, not treatment-monitoring discordance
ALTAIR/CIRCULATE-Japan	Resected CRC; n=243 randomized	No radiological disease	Tumor-informed ctDNA molecular recurrence	FTD/TPI did not significantly improve DFS vs placebo	Molecular detection does not guarantee intervention benefit	One post-adjuvant strategy

cfEBV DNA, cell-free Epstein-Barr virus DNA; CRC, colorectal cancer; ctDNA, circulating tumor DNA; DFS, disease-free survival; FTD/TPI, trifluridine/tipiracil; GU, genitourinary; HR, hazard ratio; ICI, immune checkpoint inhibitor; NSCLC, non-small-cell lung cancer; OS, overall survival; PDAC, pancreatic ductal adenocarcinoma; PFS, progression-free survival; PR, partial response; RECIST, Response Evaluation Criteria in Solid Tumors; RFS, recurrence-free survival; SD, stable disease.

### 4.4 Evidence maturity framework

To avoid conflating prediction with clinical utility, we reserve the term Level exclusively for evidence maturity: Level 1, biological association; Level 2, longitudinal prognostic validity; Level 3, multimodal incremental value; Level 4, prospective decision impact; and Level 5, prospective clinical utility. Level 5 has been achieved only in selected biomarker-specific settings and has not been established for a general discordance-aware imaging-ctDNA AI system.

## 5 Requirements for future discordance-aware multimodal AI

### 5.1 Requirements, not a validated architecture

The following functions are proposed requirements for future systems rather than capabilities established by current evidence. A clinically useful system should: (1) preserve modality-specific information; (2) model irregular longitudinal sampling; (3) classify discordance using multilabel outputs; (4) quantify uncertainty and permit abstention; (5) generate ranked biological hypotheses rather than assert causality; and (6) identify the next assessment most likely to reduce clinically relevant uncertainty.

Candidate methods include joint longitudinal and time-to-event models for linking repeatedly measured biomarkers with subsequent clinical events (18), architectures explicitly designed for irregularly sampled time series and informative missingness (19), multimodal representation learning, calibrated probabilistic prediction and conformal approaches for communicating prediction uncertainty (20), and value-of-information methods for identifying when additional information may meaningfully reduce decision uncertainty (21). Comparative evidence is insufficient to prescribe one architecture. Method selection should therefore be driven by the intended clinical use, data-generating process, interpretability requirements, and prospective validation plan.

### 5.2 Temporal alignment and missingness

Imaging and blood sampling rarely occur simultaneously. The model should ingest exact timestamps rather than force observations into artificial synchronized bins. Time since treatment initiation, time since prior assessment, and the imaging-to-blood interval should be explicit features. Missingness should be modeled rather than silently imputed when the absence of a test is clinically informative (19).

### 5.3 Modality-preserving integration

Early fusion can obscure the distinct meaning of imaging and molecular signals. Future systems should retain interpretable modality-specific representations and expose which modality contributes to a prediction or discordance label. Lesion-level imaging information is particularly important because a patient-level radiographic category can hide mixed response or oligoprogression.

### 5.4 Uncertainty, calibration, and abstention

A clinically deployed model should communicate calibrated uncertainty and should be able to abstain when data quality is insufficient, sampling is asynchronous, or predictions fall outside the validated domain (20). Decision support should distinguish low-confidence isolated mismatches from persistent high-confidence discordance.

### 5.5 Biological hypotheses require confirmation

AI cannot establish the biological cause of discordance without supporting evidence. A model may rank hypotheses such as low-shedding progression, sanctuary-site disease, emerging resistance, pseudoprogression, or technical artifact, but these remain hypotheses until supported by repeat testing, site-specific imaging, pathology, or subsequent clinical evolution.

### 5.6 Worked clinical vignette

Consider a patient with metastatic disease whose CT shows stable disease while plasma ctDNA obtained five days later rises reproducibly on two sequential assessments and a resistance-associated alteration emerges. The pattern is directional discordance with a temporal component and molecular discordance; spatial discordance remains possible because the anatomical source is unknown, and technical discordance must be assessed through assay quality and reproducibility. Because the signal is persistent and analytically credible, confidence is higher than for an isolated low-level rise. In the absence of a prospectively validated biomarker-intervention pair, the case would fall within Clinical Action Tier B-C: reassess or confirm through repeat ctDNA, earlier or lesion-directed imaging, multidisciplinary review, and biopsy when clinically justified. Automatic systemic treatment switching would not be supported.

## 6 From prediction to clinical utility: validation, actionability, and deployment

### 6.1 Clinical action tiers A-D

To avoid confusion with evidence maturity Levels, clinical responses are described as Tiers. Tier A (Observe) applies to isolated, low-confidence discordance without corroboration and supports protocol-based follow-up. Tier B (Reassess) applies to reproducible or concerning discordance that remains insufficient for intervention and supports repeat ctDNA, shortened imaging interval, or technical review. Tier C (Confirm) applies to persistent, higher-confidence discordance for which lesion-directed imaging, multidisciplinary review, or biopsy could resolve clinically important uncertainty. Tier D (Evidence-supported action) applies only when a validated disease-specific biomarker-intervention strategy supports treatment modification.

### 6.2 Defining clinical utility and potential harms

Clinical utility requires more than higher AUC or C-index. Appropriate comparators include standard imaging schedules, RECIST-based assessment, ctDNA monitoring alone, clinician judgment, and disease-specific surveillance pathways. Relevant endpoints include time to confirmed progression, diagnostic yield, appropriate treatment modification, unnecessary imaging or biopsy, progression-free and overall survival, quality of life, patient-reported outcomes, and cost-effectiveness.

Potential harms include false-positive molecular progression, lead-time bias, unnecessary imaging or invasive procedures, anxiety, financial toxicity, premature discontinuation of effective therapy, false reassurance from undetectable ctDNA, and unequal model performance across institutions, assay platforms, scanners, and patient populations. Prospective evaluation should therefore assess net benefit and patient-centered consequences, not discrimination alone.

### 6.3 Prospective validation strategy

Validation should progress from analytical and technical reproducibility to external validation, prospective decision impact, and finally clinical utility. Discordance-enriched cohorts can estimate prevalence and inter-observer reliability of taxonomy labels. Adaptive-surveillance studies can test whether model-guided reassessment improves diagnostic efficiency without excessive procedures. Randomized intervention trials are required when treatment modification is the intended use. The proposed evidence-to-clinical-translation pathway is illustrated in **Figure 3**, and the corresponding evidence-maturity levels and prospective validation requirements are summarized in **Table 3**.

**Figure 3:**
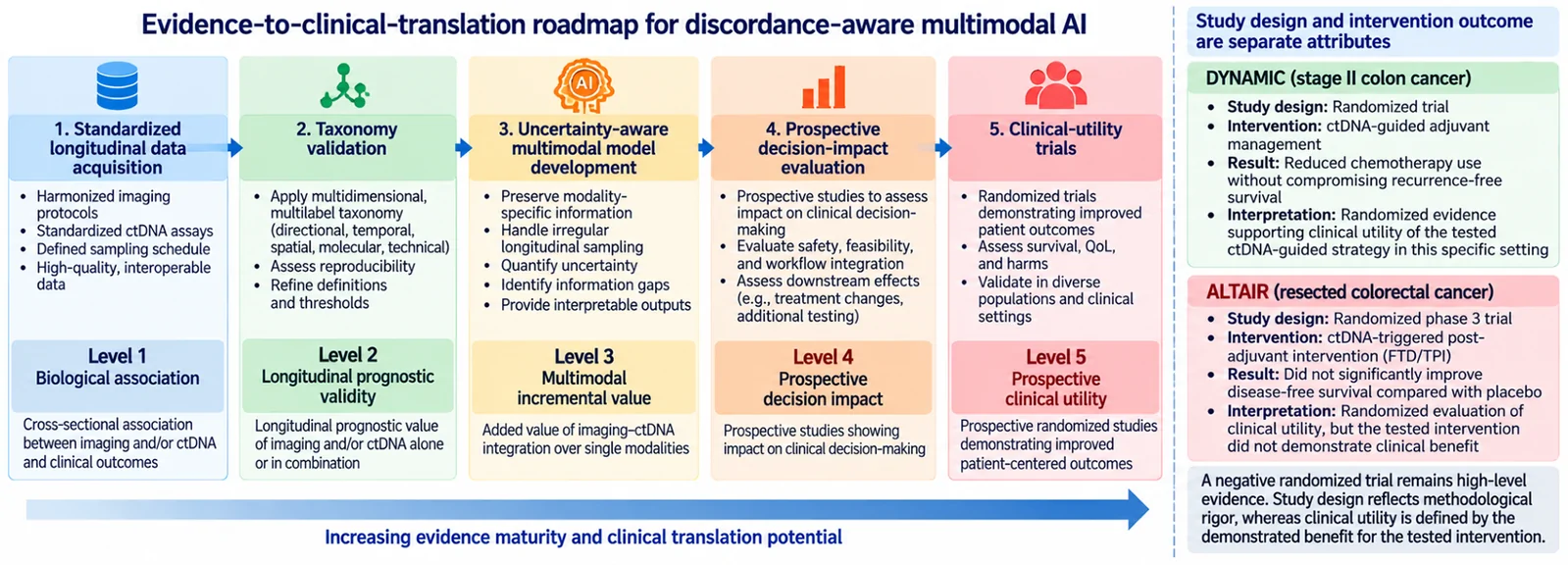
Evidence-to-clinical-translation roadmap for discordance-aware multimodal AI. Translation proceeds from standardized longitudinal data acquisition and taxonomy validation to uncertainty-aware model development, prospective decision-impact evaluation, and clinical-utility trials. Treatment modification is evaluated only when a disease-specific biomarker-intervention strategy is sufficiently supported. AI, artificial intelligence; ctDNA, circulating tumor DNA; QoL, quality of life. Initial schematic design was assisted by OpenAI ChatGPT and was reviewed and edited by the authors.

**Table 3:** Evidence maturity and prospective validation requirements for discordance-aware multimodal AI.

Evidence level	Definition	Representative evidence	Required next step	Key endpoint
Level 1: Biological association	A modality or molecular signal is associated with outcome or response.	Single-modality observational studies.	Independent replication and analytical validation.	Association strength; reproducibility
Level 2: Longitudinal prognostic validity	Serial trajectories predict subsequent clinical outcome.	Longitudinal ctDNA or imaging kinetics.	External temporal/geographic validation.	Calibration; discrimination; lead time
Level 3: Multimodal incremental value	Combined modalities add information beyond established comparators.	Imaging-ctDNA/radiomics models.	Prospective validation with prespecified comparator.	Incremental value; calibration; decision curve/net benefit
Level 4: Prospective decision impact	Model output changes diagnostic or surveillance decisions.	Prospective decision-support study.	Evaluate downstream procedures, harms, and workflow.	Decision change; diagnostic efficiency; safety
Level 5: Prospective clinical utility	Use of the biomarker/model improves patient-centered outcomes.	Established only in selected biomarker-specific contexts; not established for general discordance-aware AI.	Randomized or otherwise rigorous interventional evaluation.	PFS/OS/QoL/net benefit/cost-effectiveness

### 6.4 Deployment, governance, and generalizability

Clinical deployment raises additional requirements. Intended use and software-as-a-medical-device implications should be defined before evaluation. Data provenance, algorithm versioning, model drift, post-deployment monitoring, human oversight, accountability, privacy, interoperability, and cybersecurity should be addressed. Radiomic transportability is vulnerable to scanner, acquisition, reconstruction, and segmentation differences, whereas molecular transportability is affected by assay design, sequencing platform, bioinformatic pipelines, and evolving treatment standards.

Cost and workflow burden are also part of clinical utility. A system that recommends frequent repeat testing or additional imaging may increase resource use without improving outcomes. Deployment studies should therefore include economic evaluation, equity analyses, and performance monitoring across sites and patient subgroups. These deployment requirements should be evaluated within established reporting, standardization, and trustworthy-AI frameworks (22–29).

### 6.5 Regulatory and reporting considerations

Regulatory and reporting frameworks should be used for their intended purposes. TRIPOD+AI and CLAIM can improve transparent reporting of prediction and imaging-AI studies (22, 23); DECIDE-AI, SPIRIT-AI, and CONSORT-AI are relevant to staged clinical evaluation and interventional trials (24–26). Radiomic standardization should follow initiatives such as IBSI (27), while FUTURE-AI provides broader principles for trustworthy and deployable healthcare AI (28). Regulatory guidance for ctDNA emphasizes assay standardization, harmonization, prospective evidence, and long-term clinical outcomes when ctDNA is used in drug development (29). None of these documents validates the author-proposed discordance taxonomy itself.

## 7 Future research and prospective trial design

The next phase of the field should prioritize four linked study types. First, discordance-enriched prospective cohorts should establish the frequency, persistence, reproducibility, and biological correlates of predefined discordant states. Second, adaptive-surveillance studies should test whether multimodal signals improve the timing and efficiency of confirmatory imaging or molecular testing. Third, biology-grounded studies should use pathology, lesion-directed sampling, cerebrospinal fluid analysis where appropriate, or other orthogonal evidence to evaluate mechanistic hypotheses. Fourth, randomized trials should evaluate treatment changes only when a specific intervention is biologically plausible and supported by sufficient preliminary evidence.

Standardization is essential. Studies should report exact blood-imaging intervals, assay type and analytical characteristics, molecular metric used, imaging protocol, lesion-selection rules, missing-data handling, model calibration, uncertainty, external validation, subgroup performance, and the clinical consequences of false-positive and false-negative outputs.

The translational sequence should therefore be: classify the observed pattern, quantify uncertainty, obtain confirmation when clinically relevant, and act only when the evidence matches the proposed intervention. This sequence is intentionally more conservative than a direct pathway from discordance to treatment switching.

## 8 Critical view

Our critical view is that imaging-ctDNA discordance should be treated as a structured state of uncertainty rather than as an automatic marker of treatment failure. Previous reviews have primarily addressed the feasibility of combining imaging and ctDNA for prediction or multimodal AI more broadly (1, 2). We instead argue that the clinically important unit of analysis is the discordant trajectory itself: its direction, timing, anatomical distribution, molecular context, technical reliability, and persistence. This distinction matters because a molecular signal can be prognostic without being actionable, and higher multimodal discrimination does not establish clinical utility. Near-term AI systems should therefore prioritize uncertainty quantification, confirmation, and value-of-information support rather than autonomous treatment selection. The field should advance only when prospective studies demonstrate that discordance-aware decision support improves patient-relevant outcomes without creating excess imaging, biopsy, treatment switching, cost, or inequity. A published corrigendum to the methylation-based ctDNA tumor-fraction study clarified that patients originally classified as having an ‘unknown’ cancer type should be classified as having cancer of unknown primary (CUP) (30).

## 9 Conclusion

Longitudinal imaging and ctDNA provide complementary perspectives on tumor response, and their disagreement is neither uniformly biological nor uniformly erroneous. Quantitative studies show that discordance occurs across disease settings, but its frequency and meaning depend on tumor biology, anatomical distribution, assay performance, imaging methods, and temporal alignment. The proposed multidimensional, multilabel taxonomy is intended to make these states testable rather than to assign treatment automatically.

Current evidence supports prognostic complementarity and selected examples of biomarker-guided actionability, but it does not establish a general AI system capable of converting imaging-ctDNA discordance into treatment decisions. The most defensible near-term role for multimodal AI is uncertainty-aware decision support: preserving modality-specific information, recognizing discordant trajectories, identifying competing explanations, and recommending evidence-matched reassessment. Clinical translation will require prospective validation of net benefit, harms, transportability, governance, and cost.
