# Supplementary material for: From discordance to clinical decision support: integrating longitudinal imaging and ctDNA through multimodal artificial intelligence in precision oncology
Source: Front Oncol. 2026 Oct 27;16:1963645. doi: 10.3389/fonc.2026.1963645 (PMC13749210; doi:10.3389/fonc.2026.1963645)
Supplement: Supplementary file 1 [file Table1.docx]

| Classification dimension | Main subtype | Typical manifestation | Key explanation issue | Potential clinical actions |
| --- | --- | --- | --- | --- |
| Directional | Image stabilization, decrease in ctDNA | RECIST SD + Molecular clearance | Is it an early molecular reaction? | Continue treatment and regular follow-up examinations |
| Directional | Image stabilization, increase in ctDNA | SD + continuous molecule increase | Is it a case of subclinical progression? | Repeated ctDNA, advanced imaging |
| Directional | Imaging progression, decrease in ctDNA | PD + Molecular Reaction | Slow progression or pseudo-progression | Disease lesion-level review, considering local treatment |
| Directional | Image reduction, increase in ctDNA | PR + Molecular Progression | Is there any hidden drug-resistant clone? | Expand the assessment and consider a biopsy |
| Temporal | The ctDNA improves before the imaging does. | Early molecular decline | Is it in response to the leading signal? | Continue the treatment and verification |
| Temporal | The ctDNA progresses ahead of the imaging findings. | Molecular progress leads the way | Should intervention be carried out in advance? | Encryption monitoring should not be directly replaced with other treatments. |
| Spatial | Oligoprogression | A few lesions have progressed. | Is it a local drug resistance? | Local treatment combined with systemic treatment retention |
| Spatial | Sanctuary-site progression | Brain or pleural progression, negative plasma | Is it a low-decay area? | Specific organ examination |
| Molecular | Total quantity has decreased while drug-resistant clones have increased. | The ctDNA level decreased while new mutations increased. | Is it a cloning alternative? | Focus on qualitative rather than merely quantitative changes |
| Technical | Single isolated inconsistency | Boundary changes | Is it due to measurement error? | Repeated testing, unified platform |
