# Supplementary material for: From discordance to clinical decision support: integrating longitudinal imaging and ctDNA through multimodal artificial intelligence in precision oncology
Source: Front Oncol. 2026 Oct 27;16:1963645. doi: 10.3389/fonc.2026.1963645 (PMC13749210; doi:10.3389/fonc.2026.1963645)
Supplement: Supplementary file 2 [file Table2.docx]

| Maturity | Model task | Current typical evidence | Main output | Key defect |
| --- | --- | --- | --- | --- |
| Level 1 | Baseline complementarity | Baseline imaging biomarker + ctDNA/cfDNA | The probabilities of pCR, PFS or OS | Cannot reflect the dynamic changes during the treatment process |
| Level 2 | Early response prediction | Baseline + Detection during one early treatment session | Early reaction or residual risk | Mostly retrospective and correlational |
| Level 3 | Longitudinal risk updating | Consecutive ctDNA or imaging + treatment variables | Progress risks that change over time | Usually, unexplained modal inconsistency occurs |
| Level 4 | Discordance-aware interpretation | At present, there is very little evidence. | Inconsistent types, mechanisms and confidence levels | Lack of standardized training labels |
| Level 5 | Prospective actionability | Not yet established | Re-examination, biopsy, local or systemic treatment decisions | Lack of random or prospective clinical efficacy validation |
