# Supplementary material for: From discordance to clinical decision support: integrating longitudinal imaging and ctDNA through multimodal artificial intelligence in precision oncology
Source: Front Oncol. 2026 Oct 27;16:1963645. doi: 10.3389/fonc.2026.1963645 (PMC13749210; doi:10.3389/fonc.2026.1963645)
Supplement: Supplementary file 3 [file Table3.docx]

| Trial type | Population | AI role | Primary endpoint | Evidence generated |
| --- | --- | --- | --- | --- |
| Discordance-enriched cohort | Patients with inconsistent imaging - ctDNA results | Classification and Explanation | Consistency mechanism verification | Taxonomy verification |
| Adaptive surveillance | Patients receiving systemic treatment | Trigger encryption monitoring | Diagnosis efficiency, detection timing | AI-assisted follow-up |
| Biology-grounded validation | Biopsy can obtain samples from the patient. | Mechanism inference | Biological authenticity | Interpretable AI verification |
| AI-guided intervention | Randomized controlled trial | Decision Support | PFS, OS, QoL, cost | Clinical efficacy verification |
